# Antimicrobial Peptides in Gut Health: A Review

**DOI:** 10.3389/fnut.2021.751010

**Published:** 2021-09-30

**Authors:** Tao Gong, Jie Fu, Lexuan Shi, Xin Chen, Xin Zong

**Affiliations:** ^1^Key Laboratory of Molecular Animal Nutrition, Ministry of Education, College of Animal Sciences, Zhejiang University, Hangzhou, China; ^2^Guangzhou Dublin International College of Life Sciences and Technology, South China Agricultural University, Guangzhou, China; ^3^School of Medicine, Foshan University, Foshan, China; ^4^Key Laboratory of Animal Nutrition and Feed Science in Eastern China, Ministry of Agriculture, College of Animal Sciences, Zhejiang University, Hangzhou, China

**Keywords:** antimicrobial peptides, biological function, classification, nutrition regulation, expression mechanism

## Abstract

Animal antimicrobial peptides (AMPs), known as broad-spectrum and high-efficiency antibacterial activity, are important effector molecules in innate immune system. AMPs not only have antimicrobial, antiviral and antitumor effects but also exhibit important effects *in vivo*, such as anti-inflammatory response, recruiting immune cells, promoting epithelial damage repair, and promoting phagocytosis of bacteria. However, research on the application of AMPs is incomplete and controversial. This review mainly introduces the classification of AMPs, biological functions, as well as the mechanisms of action, expression rules, and nutrition regulation from three perspectives, aiming to provide important information for the application of AMPs.

## Introduction

The indiscriminate use of antibiotics has caused bacteria to develop resistance and super bacteria that endangers human health. Therefore, researchers are desperately searching for alternatives to antibiotics. Recently, antimicrobial peptides (AMPs) have received extensive attention because of their broad antimicrobial spectrum, low resistance to drug resistance, and no residue formation.

AMPs, also known as host defense peptides, are a key barrier in the body to prevent the invasion of foreign pathogenic bacteria. Mature AMPs generally contain 12–100 amino acid residues and have positively charged and amphiphilic molecular structures, which facilitates their interaction with the cellular targets (like negatively charged microbial membranes or others) (1). However, as studies on AMPs continue to be deepen, researchers have found that they are not omnipotent. Some bacteria can still develop resistance to AMPs, and certain AMPs can also partly kill probiotics *in vivo*. Some researchers found that when *S. aureus* was experimentally exposed to pexiganan, cross-resistance occurred (2). In this review, we summarized the classification, biological functions, active mechanism and expression of AMPs.

## Classification of AMPs

### Structural Classes of AMPs

Based on structural differences, AMPs can be roughly divided into three categories: polyamino acids, short-chain AMPs, and lipopeptides.

Polyamino acids are polymers formed by the condensation of amino groups and carboxyl groups between amino acid molecules. Cationic polyamino acids, such as polylysine, polyarginine, and polyhistidine, have bactericidal effects and have attracted a lot of attention due to their unique antibacterial mechanism and low resistance to bacterial resistance. The bacterial cell membrane can be depolarized and ruptured by polylysine through electrostatic interactions, leading to the death of bacteria (3, 4). Polyarginine contains a guanidine group, which can ionize in an alkaline environment, penetrate the outer membrane of bacteria, dissolve cells; on the other hand, bacterial contents and nucleic acids can bind to polyarginine, causing the change of replication and transcription of target cells genes (5). In acidic conditions, polyhistidine can interact efficiently with the outer membrane of anionic cells, thereby exerting an antibacterial effect (6). Short-chain AMPs are peptides that are shorter than 15 amino acids, usually chemically synthetic, and have lower production costs and optimization potential compared to other AMPs. At present, the common short-chain AMPs mainly include those that with positively charged amino acids and hydrophobic amino acids. Most short-chain AMPs commonly exhibit positive charge and hydrophobicity. While bacterial cell membranes are mostly negatively charged, which can generate electrostatic affinity with the positive charges, and easily anchored by the hydrophobicity and overall amphiphlicity of peptides, leading to the destruction of the cell membrane, outflow of bacterial contents, and eventually, death (7). Lipopeptides, amphiphilic substances, composed of peptide base connected to one or more lipid chains, usually self-assemble and form certain aggregation structures (8). Active substances can be transported into cells in the form of endocytosis through lipopeptides, and the cell walls of bacteria will also can be destroyed by lipopeptides (8, 9). Based on the shape, they can be divided into two types: cyclic lipopeptides and linear lipopeptides.

### Origin Classes of AMPs

According to different origins, AMPs usually have the following four categories: microbial AMPs, animal AMPs, plant AMPs, and other AMPs. AMPs from different origins have different antibacterial effects. Here we mainly discuss animal AMPs, which can be divided into insect AMPs, mammalian AMPs, amphibian AMPs, avian AMPs, and fish AMPs.

Insect AMPs are basic peptides with low molecular weight, good water solubility, strong thermal stability, no immunogenicity, and resistance to hydrolysis. They do not damage the normal cells of higher animals but have strong and broad-spectrum antibacterial, anticancer, and antiviral abilities (10). Mammalian AMPs, divided into defensins and cathelicidins, mainly exist in neutrophils and epithelial cells of skin and mucosa. Among them, defensins have been studied the most and constitute most of the AMPs in the AMP family. Amphibian AMPs, composed of 5–60 amino acids, usually have good water solubility, good thermal stability, and the ability to tolerate proteases. They are mostly composed of a single peptide chain, and some also have a special disulfide bond structure (11). Avian defensins are significant different from mammalian defensins in coding. Human β-defensin is encoded by only two exons, while avian β-defensin is encoded by four exons, where the first one encodes the 5'UTR region of the defensin gene, the second encodes signal and part of the precursor, the third encodes the rest of the precursor, and the third and fourth encode mature peptides (12). But AvBD12 has only three exons, which may be the last two exons fused into one during evolution (13). Moreover, in the avian cathelicidin family, the domain of the signal peptide cathelicidins are highly conserved, but the mature peptides are highly differentiated (13, 14). Fish AMPs are positively charged short-chain amino acids that participate in the host's defense mechanism. Although fish AMPs are divided into defensins and cathelicidins similar to mammals, fish also secrete specific AMPs with high salt tolerance, which might be associated with the high salt of sea water (15).

## Biological Function and Active Mechanism of AMPs

### Antibacterial Function and Active Mechanism of AMPs

**Figure 1:**
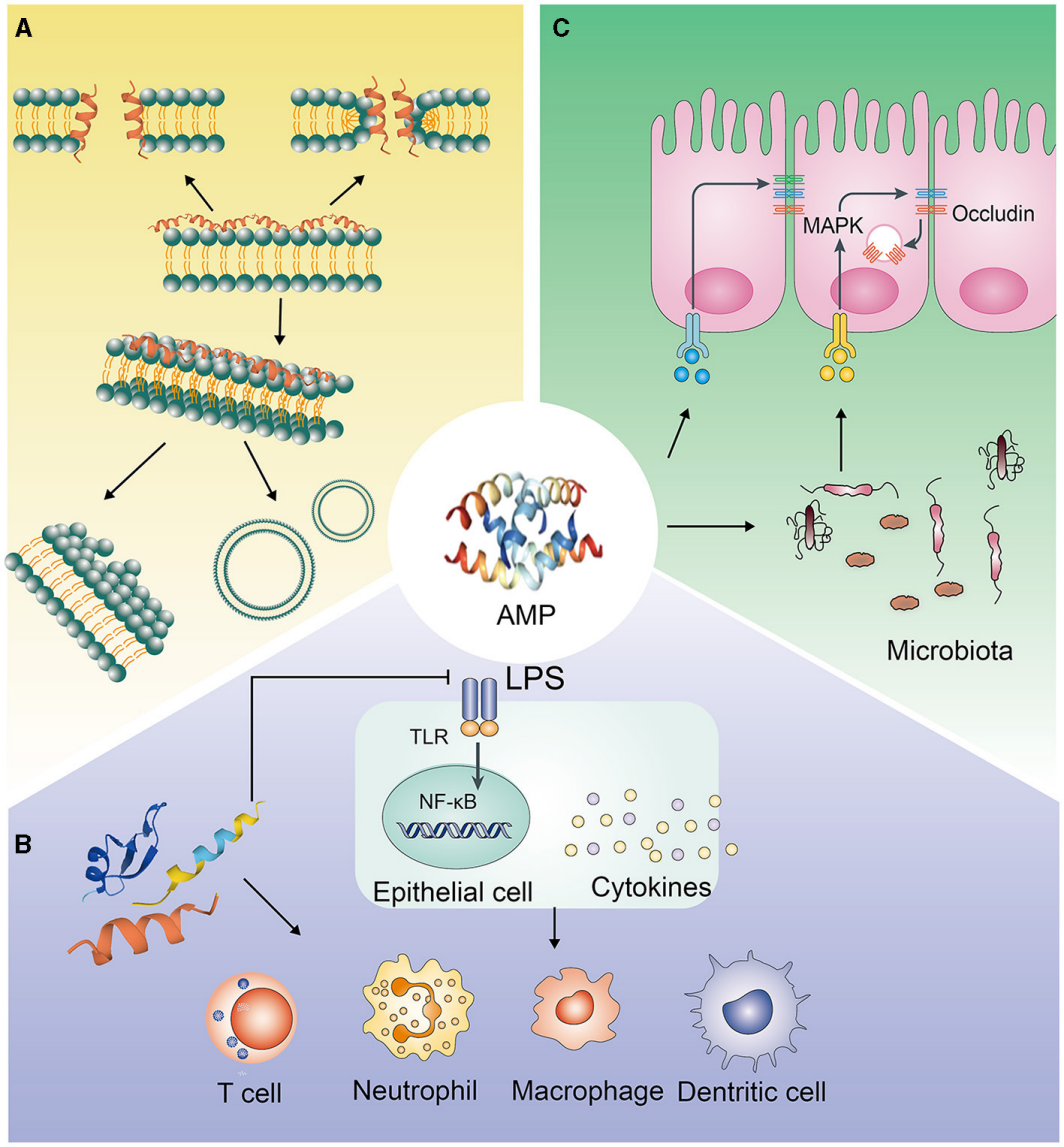
Antimicrobial pathways of AMPs. **(A)** Interacting with pathogenic bacteria directly; **(B)** Eliminating pathogenic bacteria through immune regulation; **(C)** Inhibiting the invasion of pathogenic bacteria by regulating the intestinal barrier.

AMPs have negatively charged electrostatic interactions and interactions with specific intracellular targets, which make the integrity of microbial cell membranes destroyed and the synthesis of cell proteins, DNA and RNA inhibited (16). AMPs mostly exert antibacterial effects via their secondary structure, which mainly includes α helices and β sheets. In aqueous solution, most α-AMPs are in random helical conformation, which can resume amphipathic conformations, highly structured, in membrane mimetic environment. In contrast, β-AMPs are quite more ordered in both above cases (17). It makes α-AMPs bind to bacterial membranes that the electrostatic interaction between cationic residues on the peptide and anionic lipids on the target membrane (18). β-AMPs have a linear butyl side chain, which can interact with membrane lipids through the hydrophobic subunit Bu to kill bacteria by disrupting the integrity of the bacterial membrane (19). Moreover, many studies have shown that it's essential for AMPs to ploy biological activities and primary mechanism of action that the positive charges and hydrophobic properties (Figure 1A). The electrostatic interaction, that between AMPs and the negatively charged bacterial membrane, can be promoted by the positive charges; while the bilayer of the bacterial membrane can be inserted by AMPs though hydrophobicity, which leads to the cell membrane be disrupted and permeabilitied, and the bacterial contents be leakaged and death, eventually (20–22). On the other hand, some AMPs, which can make membrane transporters blocked and cell division inhibited and can interact with nucleic acids and/or with the protein biosynthesis process, function differently and act in a non-lytic manner, preferring intracellular targets (23–25). Some membrane-active AMPs, such as indolicidin and LL-37, may penetrate the cell, interact with intracellular targets and bind to the DNA (as many cationic AMPs do) (26, 27). Other AMPs, such as the proline-rich drosocin (in the non-glycosylated form), enter the cell through transporters and act on specific intracellular proteins, without significantly perturbing its membranes (28).

The antibacterial effect of AMPs is also related to their concentration. AMPs are usually located on the surface of the cell membrane, and when the peptide concentration reaches a certain threshold, the bacterial cell membrane is destroyed (29). In locations when AMPs' physiological concentration are lower than micromolar levels, other functions such as immune regulation might be more important than killing bacterial directly. Animals generate a suite of AMPs, which act additively and often even synergistically in most cases. Hence, although the concentration of each AMP might be below the activity threshold, bacteria might still be killed by the AMP mixture (30, 31). It indicates a cooperative interaction of the AMPs molecules with the lipid monolayer that the surface pressure of the mixture is sigmoidal increasing by increasing AMPs' concentration (32). Interestingly, when the concentration is too high, AMPs may start to aggregate before attaching to the cell membrane, suggesting that their potency to disrupt the cell membrane might be reduced (33). High concentrations of AMPs were shown to increase the intestinal permeability and imbalance of intestinal bacteria in mice (34).

Research on the mechanism of action of AMPs, which resist fungi, has also been performed. Some studies have found that the intracellular targets can be affected by AMPs though ROS production, programmed cell death, mitochondrial dysfunction, disruption of cation homeostasis, ATP efflux, cell cycle impairment, autophagy, and vacuolar dysfunction. AMPs, which finish interacting with the target, can be internalized or can remain outside the fungal cell (35).

### Immunomodulatory Effects and Active Mechanism of AMPs

The AMPs' concentration in the body is lower than 2 μg/ml, which is lesser than the concentration required for bactericidal effects but is sufficient to regulate immune cell function in the physiological environment (36). Many studies have shown that immunomodulatory activity is the main biological function of AMPs (Figure 1B).

The immunomodulatory functions of AMPs mainly include: (1) Regulating the level of inflammation *in vivo*. Excessive activation and amplification of the innate immune system can cause damage to the host, and AMPs can inhibit excessive inflammation *in vivo*. The mechanisms of AMPs regulating inflammation are very complicated. LL-37 can prevent the translocation of the NF-κB subunit p65 translocation, activate MAPK and PI3K signaling pathways, and selectively upregulate the expression of anti-inflammatory factors, or directly bind to LPS, preventing its interaction with LPS binding protein, thereby inhibiting the activation of TLR4 and the downstream signaling pathways (37, 38). In B lymphocytes, mouse and human neutrophils, and dendritic cells, the abnormally high expression of proinflammatory factors, induced by LPS, can be reduced by LL-37 (36, 37, 39). Moreover, AMPs, such as C14-R1 and C12-R2, can kill bacteria via prom0oting ROS generation and causing oxidative damage (40). (2) Indirectly playing a chemotactic role by inducing or increasing the secretion of chemokines. At low physiological concentrations, AMPs can induce the chemotaxis of immune effector cells and the production of chemokines. For example, human defensins can stimulate the transcription and production of IL-8 in bronchial epithelial cells, via inducing degranulation and activation of mast cells to recruit neutrophils (37). When the physiological concentration is little higher, AMPs directly act as chemokines, recruiting granulocytes to the infection site to initiate innate and adaptive immune responses. LL-37 can mediate FPR2 receptors and CXCR2 to increase calcium efflux, thereby facilitating chemotaxis of peripheral blood monocytes and neutrophils. Moreover, LL-37 can also activate FPR2 receptors to induce monocyte chemotaxis (41, 42). Similarly, hBD-2 and hBD-3 can chemoattract monocytes through CCR2 (43). (3) Initiating and regulating specific immunity. If the innate immunity is unable to eliminate the infection, AMPs initiate and expand the host's specific immune response by signal transmission pathways, acting as a signal transduction bridge between innate immunity and specific immunity. Injection of PTd and AMP HH2-CpG can increase the secretion level of IgG by 100 times, and hence, increase the antibody level of immunoglobulin subtypes IgG2a and IgG1 (44). (4) Directly enhancing the ability to resist bacterial infections. CRAMP knockout mice are more likely to show skin necrosis due to *Streptococcal A* infection and are more likely to show urinary system infections (45, 46). (5) Activating immune cell function through specific receptors. For example, LL-37, formed a complex with DNA of host cell, activates plasma cell-like DC cells through the TLR-9 signaling pathway, making IFN-γ produced and autoimmune T cells activated (47, 48). Moreover, porcine cathelicidin protegrin-1 maintains barrier function by accelerating the migration of porcine epithelial cell, depending on the activation of insulin-like growth factor-1 receptors, to modulate immune activity (49).

### The Barrier Function and Active Mechanism of AMPs

Epithelial cells in the animal intestine, urinary tract, and respiratory tract can express AMPs. In recent years, many studies have shown that AMPs play an important role in animal mucosal and skin defense. The role of AMPs is not only to kill pathogenic bacteria but also to enhance the body's resistance to pathogenic microorganisms by enhancing the barrier function of epithelial tissue (Figure 1C). For example, LL-37 can induce the expression of various cell growth factors such as vascular endothelial growth factor and keratinocyte growth factor, stimulate the growth of intestinal epithelial cells, and ensure the integrity of the intestinal epithelial tissue (50). LL-37 can also increase the hardness of alveolar epithelial cells by interacting with fibrous actin, thereby enhancing the body's defense against *Pseudomonas aeruginosa*. Cathelicidin can increase the expression of epithelial mucins MUC1 and MUC2 via the MAPK signaling pathway and promote the repair of epithelial injury (51). Cathelicidin-WA can also promote the absorption of long-chain fatty acids by intestinal epithelial cells via the PPAR-γ signaling pathway to strengthen the intestinal barrier (52). Moreover, AMPs have also been shown to facilitate the absorption of skin-derived toxins to protect frogs from predators, as AMPs can permeabilize the epithelial tissue to enable fast transmembrane transport for co-secreted toxins (53). Meanwhile, during thermal injury, β-defensin-2 can bind to C1q to make the classical pathway inhibited, with the result that it can be reduced that the overactivation of complement cascade by human β-defensin-2 (54). Tight junctions between intestinal epithelial cells can regulate the permeability of the intestinal mucosal barrier and maintain the tightness of the intestinal epithelial cells. They are a vital part of the intestinal mucosal barrier. In recent years, studies have shown that AMPs can regulate the expression of tight junction proteins and affect the permeability of the intestinal mucosal barrier. Human defensin-1 can increase the expression of the tight junction proteins occludin and claudin-1 in epidermal keratinocytes and reduce cell permeability (55). Similarly, the AMPs, cathelicidin-BF, mastoparan X, and lactoferrin-derived peptide-20, can increase the expression of IPEC-J2 tight junction protein zonula occludens-1, occludin, and claudin-1 in rat intestinal epithelial cells, maintain the normal shape of the tight junction structure, and protect the integrity of the intestinal barrier (56–60). Moreover, the AMP microcin J25 can improve intestinal mucosal morphology and strengthen the intestinal barrier in mice, along with a reduction in intestinal permeability (34, 61). Our research results also showed that injection of PR-39 significantly alleviated the damage to the mouse intestine caused by *Salmonella* infection and dextran sodium sulfate (DSS) induction and maintained the structural integrity of the intestine and intestinal homeostasis (62). Additionally, it has been reported that AMPs can interact with intestinal microbes to regulate the structure of intestinal flora, maintain intestinal homeostasis, and strengthen the intestinal barrier (63, 64). However, AMPs such as melittin, which can open the tight junctions rapidly between cells and cause a sudden increase in cell permeability, have the opposite effect on epithelial cells (65).

## Expression and Nutrition Regulation of AMPs

### Expression of AMPs

AMPs show different expressions in different growth and developmental stages of organisms. Our research group studied the expression patterns of the β-defensin family in the intestines of Jinhua pigs and Landrace pigs of different ages and found that the expression of pBD-1 and pBD-3 genes in the intestines of piglets showed an increasing trend with the increase in age. At the age days of 20, 40, and 60, the expression levels of pBD-1, pBD-2, and pBD-3 in the intestine of Jinhua pigs were higher than those of Landrace pigs (66). Other studies have shown that weaning can significantly affect the expression of AMPs PR-39 and PG^−1^ mRNA in piglets (67). In other animals, the expression of Cathelicidin AMPs also shows developmental changes with age. The CRAMP gene of cathelicidin AMPs in the mouse intestine is highly expressed in young mice, and its expression level gradually decreases with the proliferation and differentiation of epithelial cells (68). AMPs, as the effector molecules, are significant for innate immunity. When foreign bacteria infect, the body expresses AMPs to play a corresponding immunomodulatory effect. The body has different immune responses to bacteria of different serotypes and shows selectivity. For example, *Salmonella typhimurium* can specifically upregulate the gene expression of porcine defensin-1 and defensin-2 in the epithelial cell line of the pig colon, while *Salmonella cholerae* do not stimulate the expression of AMPs (69). The infection of foreign pathogenic microorganisms and their toxins can increase the expression of β-defensin in piglets or the piglet jejunal epithelial cell line IPEC-J2 (70). For example, *S. typhimurium* can increase the gene and protein expression of PR-39 and protegrin in pig bone marrow cells, and the infection of *Streptococcus ATCC19714* can upregulate the gene expression of oral β-defensin 1 in pigs (71). We found that *E. coli* K88 infection promoted the expression of the PR-39 gene in the bone marrow, spleen, and ileum of Jinhua pigs and Landrace pigs and increased the expression of the PR-39 gene in the thymus, liver, and lung of piglets (72). The mechanism of action probably depends on the FOXO6-METTL3-m^6^A-GPR161 signaling axis (73).

### Nutritional Regulation of AMPs

It is more economical and effective to regulate the expression of endogenous AMPs through nutritional means, among which, VD3 and butyric acid are the most important regulators. VD3 can induce the expression of AMPs in a variety of human cell lines and primary cells (74). VD3 can inhibit bacteria by killing them through the effect of LL-37 and the maturation of phagosomes; additionally, it can induce the expression of HBD-2 and has a dose-dependent effect (68). Retinoic acid, metabolized by VA, can induce the expression of PR-39 in pigs (70). Additionally, bacterial polysaccharides can increase the expression levels of PR-39 in the bone marrow and liver and hepcidin in the liver (75). Arginine, isoleucine, leucine, and valine can modulate the expression of intestinal endogenous β-defensins in porcine through multiple pathways (76, 77).

## Prospects

To minimize the excessive use of antibiotics, the emergence of AMPs might solve key problems such as bacterial resistance and drug residues. However, the application of AMPs in the aquaculture industry still faces many challenges. With the continuous advancement in science and technology, the technical barriers to the application of AMPs in the aquaculture industry might be gradually removed. By constructing a suitable expression system and improving the expression strategy, many recombinant AMPs with low cost, high yield, and excellent activity can be obtained. Through genetic and artificial modifications, the antimicrobial function of AMPs can be maximized. The use of amidation, cyclic methods such as D-amino acid substitutions, and coating can improve the stability and safety of AMPs in the body. A comprehensive analysis of AMPs from the structural features and pharmacokinetics to the immunomodulatory mechanisms is benefit to the development of safer and more efficient AMPs for health. In addition, with the deepening of molecular directed evolution and systematic molecular evolution research in AMPs, more high-quality AMPs will be gradually developed, and the application of AMPs will be more and more extensive.

## Author Contributions

XZ and XC: designed the study. TG and JF: wrote the manuscript. XZ, TG, and LS: critically made comments to the manuscript. All authors have read and agreed to the published version of the manuscript.

## Funding

This study was supported by the National Natural Science Foundation of China (key program, 31630075, 32002185), the Zhejiang Provincial Natural Science Foundation of China (Grant No. LQ21C170002), and the Guangdong Basic and Applied Basic Research Fund Project under grant (number 2019A1515110092, 2019A1515110696).

## Conflict of Interest

The authors declare that the research was conducted in the absence of any commercial or financial relationships that could be construed as a potential conflict of interest.

## Publisher's Note

All claims expressed in this article are solely those of the authors and do not necessarily represent those of their affiliated organizations, or those of the publisher, the editors and the reviewers. Any product that may be evaluated in this article, or claim that may be made by its manufacturer, is not guaranteed or endorsed by the publisher.
